# Developing and evaluating an instrument to measure Recovery After INtensive care: the RAIN instrument

**DOI:** 10.1186/s12912-018-0275-1

**Published:** 2018-02-12

**Authors:** Ingegerd Bergbom, Veronika Karlsson, Mona Ringdal

**Affiliations:** 1 Institute of Health and Care Sciences at the Sahlgrenska Academy, University of Gothenburg, Gothenburg, Sweden; 2 Faculty of Caring Science, Work Life and Social Welfare, Borås University, Borås, Sweden; 3 Department of Health Sciences, University West, Trollhättan, Sweden; 4 Department of Anesthetic and Intensive Care, Kungälvs hospital, Kungälv, Sweden

**Keywords:** Recovery, Intensive care recovery, Factor analysis, Recovery questionnaire

## Abstract

**Background:**

Measuring and evaluating patients' recovery, following intensive care, is essential for assessing their recovery process. By using a questionnaire, which includes spiritual and existential aspects, possibilities for identifying appropriate nursing care activities may be facilitated. The study describes the development and evaluation of a recovery questionnaire and its validity and reliability.

**Methods:**

A questionnaire consisting of 30 items on a 5-point Likert scale was completed by 169 patients (103 men, 66 women), 18 years or older (m=69, SD 12.5) at 2, 6, 12 or 24 months following discharge from an ICU. An exploratory factor analysis, including a principal component analysis with orthogonal varimax rotation, was conducted. Ten initial items, with loadings below 0.40, were removed. The internal item/scale structure obtained in the principal component analysis was tested in relation to convergent and discrimination validity with a multi-trait analysis. Items consistency and reliability were assessed by Cronbach's alpha and internal item consistency. Test of scale quality, the proportion of missing values and respondents' scoring at maximum and minimum levels were also conducted.

**Results:**

A total of 20 items in six factors - forward looking, supporting relations, existential ruminations, revaluation of life, physical and mental strength and need of social support were extracted with eigen values above one. Together, they explained 75% of the variance. The half-scale criterion showed that the proportion of incomplete scale scores ranged from 0% to 4.3%. When testing the scale's ability to differentiate between levels of the assessed concept, we found that the observed range of scale scores covered the theoretical range. Substantial proportions of respondents, who scored at the ceiling for forward looking and supporting relations and at floor for the need of social support, were found. These findings should be further investigated.

**Conclusion:**

The factor analysis, including discriminant validity and the mean value for the item correlations, was found to be excellent. The RAIN instrument could be used to assess recovery following intensive care. It could provide post-ICU clinics and community/primary healthcare nurses with valuable information on which areas patients may need more support.

## Background

Many aspects of health and recovery have been measured and evaluated in relation to different healthcare areas as well as in relation to certain treatments or diagnoses. Different questionnaires and instruments have been developed to measure or predict recovery time following hospital care and illnesses [1–8] focusing on different dimensions, such as cognition, physical symptoms, anxiety, depression, quality of life and health. However, spiritual and existential thoughts have not been a focus in these measurements on the recovery process. After being discharged from intensive care unit (ICU) and usually a life threatening medical condition, patients’ lives may include not only lingering physical discomfort and difficulties in daily life but also thoughts about life and death and their future. Therefore, these aspects, when estimating patients’ recovery following ICU care and experiences of having been seriously ill/injured, are of importance for planning and implementing care actions.

Warrén Stomberg et al. [9] have, in a literature review, described ten recovery questionnaires/instruments that assess or measure recovery following day surgical procedures. The assessed dimensions were divided into two groups: 1) physiological-physical and 2) emotional, nutritional, eliminating, nauseous and vomiting. In a systematic review by Ebrahim et al. [10], they found that 44 studies indicated that patients’ positive recovery expectations predicted a quicker return to work while negative expectations predicted longer sick leave. However, few studies used a psychometrically valid instrument for measuring these recovery expectations.

Recovery following serious illness/injury and ICU care has been described and investigated in several studies, but there is no valid instrument for measuring recovery that include existential and spiritual health. Recovery has been evaluated from one to several years following IC [11–14] or during a period immediately following discharge from the hospital [15]. Measuring health related quality of life (HRQL) has been one method to estimate patients’ recoveries [12, 13]. Discomforting delusional memories [12, 14] have been found in patients after hospital and ICU discharge. Kelly and McKinley [16] reported that patients, six months following discharge, still suffered from mobility difficulties and sleep disturbances. Muscle weakness and loss of body weight have also been reported following critical illness and ICU care [17–20]. In an Australian qualitative interview study, six months following ICU [21] most patients reported having returned to a healthy state even if they continued to report pain, sleeping disturbances, tiredness, depression, feeling of loneliness, and financial problems. Post-Traumatic Stress Disorder (PTSD), as a complication following critical illnesses and IC, has also been reported in several studies since the 1980s [19, 22–25]. Based on such research, Åkerman et al [26] developed the 3-SET 4P questionnaire for evaluating recovery after ICU, which focused on physical and psychosocial problems but not existential or spiritual issues. The questionnaire consists of 53 items on a 5-point Likert scale with 16 physical, 26 psychosocial, and 11 follow-up care items. In the pilot study, 39 patients answered the questionnaire, and 17 of those did the retest. Physical problems revealed four factors (11 questions), psychosocial five factors (22 items), and follow-up four factors (10 items). They concluded that patients following IC described several discomforts, disabilities, and symptoms during the recovery process.

### The recovery concept and theoretical framework

The word recovery consists of “re” and “cover”, which could have three meanings: 1) recovery (from something) – the process of becoming well again after illness/injury; 2) recovery (in something) – the process of improving or becoming stronger again; 3) recovery (of something) – the action or process of getting something back that has been stolen or lost. As each of these meanings contain a process, an aspect of time is inherently involved [27]. Based on these meanings recovery and experiences of health, well-being, as well as quality of life can be seen as both connected and interrelated. According to Gadamer [28], health is connected to the rhythm of life: breathing, sleeping, and metabolic processes. When equilibrium is lost in these, it is not only a medical-biological state but also a social and life-historical transformation the patients go through as they will no longer return to the same people they were before falling ill. Loss of health and illness itself may also be a loss of one’s physical and/or mental freedom, which affects life as a whole, evoking existential issues. Therefore, recovery could be seen as the challenge to restore one’s own sense of self-identity. Based on the thought that recovery and health are interrelated, recovery could be viewed as a movement from illness towards health, involving both objective and subjective dimensions [29]. Objective dimensions contain symptoms and signs that can be assessed by other people, such as physicians and nurses. Subjective dimensions contain self-reported experiences or feelings. From the patient’s perspective, the experiences and feelings of being recovered or feeling well or not is of interest, as this may affect their need for care and support/help. People’s experience of health and recovery always includes their perception based on their previous experience and personal values on what quality of life means.

Recovery and regaining health can be seen as a movement from disintegration towards integration and of wholeness [29, 30]. Integration means coordinating separate parts into something more functional wholeness, where the parts remain qualitatively separated. The wholeness is seen as multidimensional and consists of the individual and his/her whole environment, where the meaning of life, life motivation, will and a perspective for a future is vital. The word “integrate” means to make whole. Being recovered or being healthy is understood as an integrated condition of freshness, soundness and well-being. Disintegration is the opposite, which means dissolution [29, 30]. Issues concerning the patients’ experiences of their illness, the care event and maybe lingering symptoms and discomfort have been integrated into their lives and, in some sense, been given meaning. For example patients claim that they feel well, despite reporting discomforting symptoms. They can claim that they could live with these discomforts, and that they feel lucky to have survived [20]. Thus, a movement towards integration means becoming “whole” again, re-covered, where the suffering becomes bearable. This means being able to look forward, to yearn, to be open and ready to interact with others, give or find meaning in the past and present. These issues refer to spiritual or existential dimensions of recovery. In terms of this study, we define recovery as a process towards integration or wholeness even if setbacks are experienced.

It can be concluded that health and recovery are complex phenomena and, therefore, difficult to measure [28]. There are several areas, such as spiritual, existential, and social, that are not covered in many health and recovery questionnaires. A questionnaire that takes these issues in consideration could be useful in clinical follow-up care of patients who have been cared for in the ICU, but it could also be a useful tool for the patients themselves to evaluate changes throughout their recovery process.

### Objectives

The aim was to describe the development and evaluation of the Recovery after Intensive Care questionnaire’s (RAIN) validity and reliability.

### Development of the questionnaire

Based on previous research on patients’ recovery following ICU care [11–13, 31, 32], patient interviews [20] and the thoughts and ideas about integration, health and recovery [28, 30], basic elements in the recovery process and condition were identified by the authors. These elements were: 1) bodily – when the body has returned to an acceptable condition, and it works as it should again, thus regaining a form of freedom; 2) mentally-socially – when a person transitions from being excluded from the world of healthy people to becoming included by reaching out and interacting; 3) existentially – when a normal daily world, including routines, has developed and a revaluation of life takes place, reviewing the past but also looking forward to the future by evaluating what is meaningful; and 4) Spiritual – when they have the will to live again, are looking forward or longing for something, and are also able to share their own experiences in order to cope with their pain or suffering. Based on these elements, 30 items were constructed by the authors.

In the next phase, these items were evaluated and analyzed by an independent expert group of four faculty members. The criteria for inviting these experts were that they should be registered nurses, have a specialist nurse education in intensive care and be experienced in caring for patients in ICU before, during or after serious/critical/acute illness/injuries. At least one of them should have conducted research in ICU. These experts evaluated the items to determine if they were clear and easy to understand and items logical order. This resulted in four open ended and two yes/no questions being added. In the end, the questionnaire consisted of 36 questions, and of these, 30 were on a Likert type scale.

### Patient pilot-test and evaluation of reliability and validity

In phase three, a pilot test of the questionnaire (36 items) was conducted. Four patients were recruited by one of the authors and another four patients were recruited by an IC nurse to participate. All patients were recruited six months after discharge from two hospital ICUs in Sweden. Thus, a total of eight patients of different ages and gender answered the questionnaire. They were asked to judge and assess the questions’ relevancy, clarity, and ease of answering. One patient reported that the two questions about the relationship to relatives and sharing thoughts with relatives were repetitive. All patients had the opinion that the questions were easy to answer.

### Content validity test

In the fourth phase, seven faculty members, who were experts within healthcare and/or IC were invited to evaluate and assess the relevancy and clarity of the 36 items using a dichotomous scale. At this meeting, one of the authors had asked one of the attendants to be in charge of the meeting. A content validity index (CVI) [33] was conducted and found to be acceptable.

### The final questionnaire

The final questionnaire consisted of a total of 36 items. The 30 Likert type questions, ranging from 1-No/Never to 5-Yes, very much/often, meaning that the higher the number, the higher recovery, constituted the foundation for testing the instrument’s properties. The four open-ended questions about present discomforts or symptoms, which symptom or discomfort was the most troubling, what the patient was longing for, and how the patient felt about their situation at the moment in relation to the reason for needing IC were excluded when analyzing the answers. The two financial questions were Yes/No, and they were considered part of the demographic data and thus not included in the analysis. Thus, the instrument RAIN consists of 30 items.

### Participants and procedure

Patients were recruited consecutively by a critical care nurse (CCN) from an eight-bed general ICU in a county hospital in Sweden. In this ICU, patients were treated for surgical and medical conditions and trauma. The study inclusion criteria were that all patients were at least 18 years old, had received care at the ICU for at least 24 hours, and could read and write in Swedish. The CCN from the post-ICU patient reception and another CCN phoned eligible patients consecutively around one month after being discharged from the ICU and asked if the researchers could call them regarding the study. At the same time, the patients were informed about the study. The patients were then phoned by the researchers and invited to participate in the study, which would require them to answer a questionnaire. The authors obtained the patients’ addresses, and then sent the questionnaire package, which included RAIN, a prepaid envelope, a written consent letter, and information about the study. The patients were to answer the questionnaire and send the questionnaire and informed consent letter back. The questionnaires were sent out approximately 2, 6, 12, or 24 months after the patient had left the ICU. The reason for evaluating and measuring recovery at different points of time was that recovery has a time aspect, meaning that patients may adapt to any lingering discomfort over a period of time. Prior to each mailing, the authors contacted the national registration authority to ensure that the patient was alive and the address was valid. The data were collected between 2013 and 2016. This length of time period was due to the fact that we wanted to investigate recovery even one and two years after ICU discharge. This meant that the questionnaire was sent 1-2 years after patients had agreed to participate in the study.

### Ethical approval

The Regional Ethical Review board at the University of Gothenburg approved the study (Dnr 695-10, 2010) before data collection commenced.

## Methods

### Analyses of factor structure

**Fig. 1:**
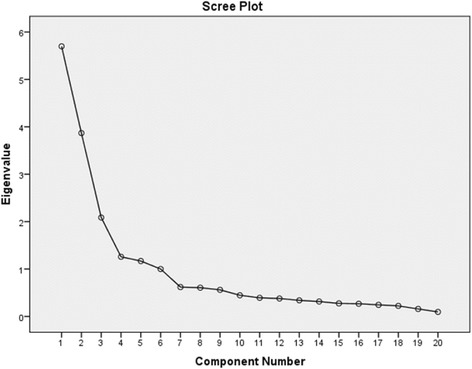
A scree plot showing factors (component number) above one in eigenvalue

Initially, a potential factor structure of the 30-item scale was tested by means of exploratory factor analysis. The method used was a principal component analysis with orthogonal varimax rotation [34]. All statistical analyses were run on PASW/SPSS Statistics version 22. More items were removed based on this principal component analysis. Thus, items with low loadings (below 0.40) across all the suggested factors as well as items that loaded equally on several factors were removed. Overall, this resulted in the removal of 10 items. The remaining 20 items were thereafter analyzed a second time with the same type of principal component analysis. In this second analysis, three criteria for factor extraction were applied: Eigen values above one, the scree plot and homogeneity, and meaningfulness of items building up each factor (Fig. 1).

A multi-trait analysis performed after the final principal component analysis demonstrate construct validity of the scale in relation to convergence and discrimination. These tests could be regarded as a simple form of confirmatory factor analyses to measure latent factors [34, 35].

Convergent validity demonstrate the related stability in the scale. It was tested by a correlation between items and the expected scale corrected for overlap, where the correlation should be more than 0.40^36^ to show convergent validity. The scale was also tested for discriminant validity by taking the items that correlated higher with the hypothesized scale compared to all other scales.

Item consistency and reliability were further assessed by internal item consistency and Cronbach’s alpha. According to conventional rules, the Cronbach alpha coefficient should exceed 0.70 [34]. Internal item consistency (Table 2) should not be lower than 0.40 [36]. Both of these values were reached for all items that remained in the questionnaire.

The quality of a scale also depend on maximum (ceiling) or minimum (floor) levels of respondents scoring. Furthermore the proportion of missing values should be taken into consideration. The half-scale criterion was used to handle missing items within a scale [35]. Thus, if a respondent answered all items connected to a scale, a sum score was calculated to form the scale score. If a respondent answered 50% or more (but not all) of the items, a mean value of the answered questions was calculated. This mean value was then imputed to form a sum score of all missing items. Finally, if a respondent answered fewer than 50% of the items connected to a scale, the missing values were treated as missing.

## Results

During a period of three years in one ICU, 169 patients answered the questionnaire. Of the 169 respondents, 61% (103) were male, 39% (66) were female, and the mean age was 69±12.5 years. Regarding their financial situation, 78% of the patients reported that their situation was acceptable, but 31% found that it had worsened since their critical illness and stay in the ICU.

### Exploratory factor analysis

The principal component analysis performed on the 20 remaining items showed a very clear factor structure (see Table 1). All items loaded satisfactorily high and clear on a corresponding factor. Table 1 shows the rotated pattern matrix from the analysis. Six factors were extracted with eigen values above one, and together they explained 75% of the variance of the 20 items. The factors were labelled: forward looking, supporting relations, existential ruminations, revaluation of life, physical and mental strength, and need of social support.

**Table 1:** Results of the Principle Component Analysis and factor loading of the 20-item Version of the RAIN instrument

	Forward looking	Supporting relations	Existential ruminations	Revaluation of life	Physical and mental strength	Need of social support	
Can you look forward?	.866						
Feel hope for the future	.862						
Plan for the future	.806						
Prepare to go forward	.680						
Share thoughts with relatives		.887					
Energy to be together with relatives		.885			.328		
Share thoughts about critical illness		.803					
Someone to speak to about difficulties		.617					
Thoughts about the closeness to death			.830				
Thoughts about your critical illness			.828				
Thoughts about your disease			.770				
Thoughts about hospital stay			.729				
Revalue life				.897			
Appreciate different things in life				.869			
Discover new characteristics of yourself				.716			
Recovered physically	.411				.746		
Recovered mentally					.719		
Sleeping well					.700		
Talk to someone outside of your family						.878	
Need to talk to a professional						.836	

### Tests of internal structure, reliability, and scale properties

The results of these tests are displayed in Table 2. All 20 items surpassed the criterion for satisfactory convergent validity, i.e., item-scale correlations > 0.40 when corrected for overlap. The mean values for these correlations ranged from 0.57 to 0.78, and no separate correlation was lower than 0.53. The Cronbach alpha coefficient, a related measure of item consistency, was accordingly very good for all scales, ranging from 0.75 to 0.90. Likewise, the tests of item discriminant validity also revealed satisfactory results.

**Table 2:** Summary of Multi-trait Scaling Analyses of the RAIN-instrument (N = 169)

	Forward looking	Supporting relations	Existential ruminations	Revaluation of life	Physical and mental strength	Need of social support
Number of items	4	4	4	3	3	2
Number of scale levels	16	16	16	12	12	8
Theoretical range	4-20	4-20	4-20	3-15	3-15	2-10
Observed range	4-20	4-20	4-20	3-15	3-15	2-10
Mean (*SD*)^a^	16.12 (3.8)	16.96 (3.6)	12.41 (3.7)	9.12 (3.8)	11.73 (2.9)	4.10 (2.5)
% incomplete scale score^b^	4.3	1.1	2.4	4.3	0.0	3.7
% at ceiling	19.6	32.3	2.5	5.7	15.2	5.1
% at floor	1.3	1.3	1.9	12.7	0.6	48.1
Mean (R) internal consistency^c^	0.78 (.68-.89)	0.72 (.59-.80)	0.66 (.58-.71)	0.68 (.61-.72)	0.57 (.52-.64)	0.73 (.73-.73)
Item-scale discriminant validity^d^	0/0/0/100	0/0/10/90	0/0/0/100	0/0/0/100	0/0/13/87	0/0/0/ 100
Cronbach’s α	0.90	0.86	0.84	0.83	0.75	0.82
% calculated scale scores^e^	1.2	5.3	0.6	0.6	1.2	0.6

^a^Mean(*SD*) of summed scores.

^b^Missing according to half scale criterion.

^c^Pearson correlations between items and hypothesized scale, corrected overlap. Mean of correlations for each scale and range.

^d^Percent correlations that are significantly lower/lower/higher/significantly higher with hypothesized scale compared to other scales.

^e^Calculated according to the half-scale criterion

Another examined scale property was the proportion of incomplete scale scores. When using the half-scale criterion, the proportion of scales scores treated as missing ranged from 0 percent to 4.3 percent, and the proportion of calculated scale scores ranged from 0.6 percent to 5.3 percent (see Table 2). Furthermore, there were no single items that clearly stood out as considerably more “missed” than others. Out of the 169 respondents, the frequency of non-answered items ranged from zero (one item) to eight (two items). Together, these data imply that the respondents in general understood and were able to respond to all items.

Further tests concerned the observed range of scale scores compared with the theoretical range. An important property of a scale is its ability to differentiate between levels of the assessed concept. The extent to which observed scale scores cover the theoretical score range is an indication of that ability. As can be seen in Table 2, the theoretical range was very well covered by the observed scale scores.

The percent of responses at the floor and ceiling levels showed the proportion of responses at the extreme endpoints of the scales. Substantial proportions of respondents who scored at the ceiling were found for the scales labelled forward looking (19.6%) and supporting relations (32.3%). The opposite was found for the need of social support scale. Here, 48.1% scored at the floor. Such high ceiling/floor effects could depend on two aspects. First, the study sample could be among those who have recovered best, are forward looking, and have little need for further support. Second, the scales in question could be less efficient at discriminating on the more positive ends of the ability to look forward and the need for further social support.

## Discussion

This study aimed to describe the development and evaluate the validity and reliability of a new instrument to measure recovery among patients after ICU treatment. As mentioned earlier, several instruments measuring postoperative recovery have been developed and used in many countries, but no instrument for post ICU care recovery which includes existential questions has been available to the best of our knowledge. Evaluating and measuring recovery following IC is complicated as many factors may influence the patients’ own opinions about their recovery. In post-surgical and ICU follow ups, HRQL has been used in order to measure patients’ opinions on their quality of life [12, 13]. For the recovery instruments that are used, most have focused simply on physical symptoms and mental issues, omitting many other facets of recovery.

The questions in the RAIN questionnaire were based upon previous empirical research, thoughts and theory models of health as a feeling of wholeness, where body, soul and spirit are in unity [30]. Moreover, the questionnaire was developed upon the understanding that recovery is seen as a process of regaining health, a movement towards integration even if setbacks sometimes occur. Therefore, dimensions that also reflect existential, social and spiritual life issues were seen as important as previous studies have stated these aspects impact patients’ recoveries [11–13, 20, 32]. For example, patients expressed that they felt well but, at the same time, were in need of help from family members [20] or suffered from thoughts of death and could not look forward to anything in life [31]. This might reflect thoughts about life and what life means, which Cöster [37] describes as ‘*livsförståelse,*’ meaning understanding of life/to view life. If life has no meaning, there is no health or well-being [38]. Such thoughts are also expressed in Morse’s theory Responding to Threats to Integrity of Self [39].

The analysis resulted in six clear factors. This supports the idea that recovery depends on several factors, not only physical symptoms and discomforts. Such factors are close relationships, thoughts and beliefs, and what is seen as important and meaningful in life. A wide range of issues are represented in the instrument, and the discriminant validity and the mean value of the item correlations found satisfactory results, suggesting this new scale could prove useful to measure recovery in patients who received IC treatment.

The dimension “supporting relations” contained four items concerning issues that showed the importance of sharing thoughts and experiences with relatives and/or others. This is in line with findings in a study by Ringdal et al [40]. It was found that all concern and care given by family members as well as healthcare professionals made the patients feel accepted even if they were in need of help and felt weak. If recovery is facilitated by patients sharing their thoughts and experiences with others, nurses and physicians might either ask about patients’ thoughts and experiences or encourage relatives and family members to listen to their loved ones concerns. Experiences from critical illness/injury, ICU treatment and care can be seen as belonging to each individual’s life history. Ringdal and Rose [41] discuss that patients’ discomforting memories belonging to the ICU period as well as difficulties to remember parts of their lives may affect their present and future life and thus recovery.

It was found that most patients answered that they did not need to talk to any professionals about their thoughts and feelings. It could be discussed if this question “Do you need to talk to a professional?” evokes thoughts about the need of psychological or psychiatric expertise rather than another medical professional. Also, the question, “Do you need to talk to someone outside the family?” may further indicate that illness, suffering, and recovery from illness/injury are seen as a private matter. The need of talking and sharing thoughts about the illness/injury and the ICU stay could be considered exclusively a family/relative concern as the mean value for factor two about the family is relatively high. These factors could be influencing the results.

Other explanations may also be connected to the fact that those patients that agreed to participate in the study may have had a successful recovery and were probably feeling relatively well. Patients who did not accept the invitation may have felt worse or did not have the strength to participate, potentially skewing our results. Unfortunately, we do not have any statistics on how many denied participation or how many were not reachable; therefore, we cannot determine the response rate. This could be seen as a limitation when assessing the recovery of the population. However, in this study, our focus was to describe the questionnaire and its properties and not the recovery of the 169 patients at this early stage of questionnaire development.

Despite these limitations, the participants were heterogeneous and represented both genders, a wide range of ages, and lapsed time, after ICU discharge. The sample size (N = 169), in relation to the number of the 30 items, met the recommended criteria of five respondents for each item [42].

There were few missing items, which indicate that either the questions were not difficult to answer or were all found relevant enough to be answered. The conceptual framework with a six-factor solution explained 75% of the variance, which is satisfactory. However, the ceiling and floor levels must be investigated in future studies to determine their impact on the overall scale. The floor and/or ceiling effects can result because of two circumstances: 1) the study group was not representative in some way by either containing patients that were too sick or too healthy. If this is the case, the questionnaire can only be tested with a new study with new patients. 2) The scale cannot cover all aspects, and this can only be tested by adding items that cover the shortcomings of the scale. This will be tested in a future study where we will continue this project and then add items to avoid potential floor and ceilings effects.

While the RAIN instrument provides an opportunity to quantify recovery, it also provides nurses with useful and valuable information for follow-up communications with former patients at so called post-ICU clinics. The RAIN instrument can also facilitate for primary and community healthcare nurses to communicate and follow-up with patients who have been seriously ill/injured and cared for at ICUs.

## Conclusion

The factor analysis including discriminant validity and the mean value of the item correlations were found satisfactory. The Cronbach alpha coefficient, a related measure of item consistency, was accordingly very good for all scales, ranging from 0.75 to 0.90. Based on these findings we recommend nurses and/or caregivers use the RAIN instrument for follow-up or post-ICU services on patients who received intensive care to get a more holistic view of their recovery.

## References

[1] Royse CF, Newman S, Williams Z, Wilkinson DJ (2013). A human volunteer study to identify variability in performance in the cognitive domain of the postoperative quality of recovery scale. Anesthesiology.

[2] Lizana FG, Bota DP, De Cubber M, Vincent J-L (2003). Long-term outcome in ICU patients: What about quality of life?. Intensive Care Med.

[3] Lippa SM, Lange RT, Bailie JM, Kennedy JE, Brickell TA, Psych D, French LM (2016). Utility of the Validity-10 scale across the recovery trajectory following traumatic brain injury. JRRD.

[4] Allvin R, Svensson E, Rawal N, Ehnfors M, Kling A-M, Idwall E (2011). The Postoperative Recovery Profile (PRP) – a multidimensional questionnaire for evaluation of recovery profiles. J Eval Clin Pract.

[5] Meuser KT, Gingerich S, Salyers MP, McGuire AB, Reyes RU, Cunningham H (2004). The illness management and recovery (IMR) scales. (Client and Clinician version).

[6] Myles PS, Weitkamp B, Jones K, Melick J, Hensen S (2000). Validity and reliability of a postoperative quality of recovery score: the OoR-40. Brit J Anaesthesia.

[7] McIntosh S, Adams J (2011). Anxiety and quality of recovery in day surgery: A questionnaire study using Hospital Anxiety and Depression Scale and Quality of Recovery Score. Internat J Nurs Pract.

[8] Hancock N, Newton SJ, Honey A, Bundy AC, O´shea K. Recovery Assessment Scale – Domain and Stages (RAS-DS). Its feasibility and outcome measurement capacity. Aust NZ J Psychiatry. 2015;49:624-633. 10.1177/0004867414564084 PMC4941096 25526940

[9] Warrén Stomberg M, Saxborn E, Gambreus S, Brattwall M, Jakobsson JG (2015). Tools for the assessment of the recovery process following discharge from day surgery: a literature review. Clinical Feature.

[10] Ebrahim S, Malachowski C, Kamal el Din M, Mulla SM, Montoya L, Bance S, Busse JW (2015). Expectations of one’s own recovery. Measures of patients’ expectations about recovery. A systematic review. Journal Occup Rehab.

[11] Olsson U, Bosaeus I, Bergbom I (2010). Patients´ experiences of the recovery period 12 months after upper gastrointestinal surgery. Gastroenterology Nurs.

[12] Ringdal M, Plos K, Örtenwall P, Bergbom I (2010). Memories and health related quality of life after intensive care – a follow-up study. Crit Care Med.

[13] Pettersson M, Bergbom I, Mattsson E (2012). Health related quality of life after treatment of Adominal Aortic Aneurysm with open repair and endovascular techniques – a two-year follow-up. Surg Sci.

[14] Zetterlund P, Plos K, Bergbom I, Ringdal M (2012). Memories from Intensive Care unit persists for several years – A longitudinal prospective multi-centre study. Intensive & Crit Care Nurs.

[15] Glimelius Pettersson C, Ringdal M, Bergbom I. Diaries and memories following an ICU stay; a 2-months follow-up study. Nurs Crit Care. 2015; 10.1111/nic.12162. 10.1111/nicc.12162 26010232

[16] Kelly AM, McKinley S (2010). Patients’ recovery after critical illness at early follow up. J Clin Nurs.

[17] Herridge MS, Cheung AM, Tansey CM (2003). One year outcomes in survivors of the acute respiratory distress syndrome. NEngl J Med.

[18] Bercker S, Weber-Carstens S, Deja M, Grimm C, Wolf S, Behse F (2005). Critical illness polyneuropathy and myopathy in patients with acute respiratory distress syndrome. Crit Care Med.

[19] Deacon K (2012). Re-building life after ICU: A qualitative study of the patients’ perspective. Intensive & Crit Care Nurs.

[20] Karlsson V, Bergbom I, Ringdal M, Jonsson A (2016). After discharge home: a qualitative analysis of older ICU patients´ experiences and care needs. Scand J Caring Sci.

[21] Daffurn K, Bishop GF, Hillman KM, Bauman A (1994). Problems following discharge after intensive care. Intensive & Crit Care Nurs.

[22] Kuch K, Swinson RP. Post-traumatic stress disorder. In: Vincent J-L, editor. Updates in intensive care and emergency medicine. Berlin: Springer-Verlag, Berlin 1988. p. 548-555.

[23] Jones C, Griffiths RD, Macmillan RR, Palmer TEA (1994). Psychological problems occurring after intensive care. Brit J Intensive Care..

[24] Schandl A, Brattström O, Svensson-Raskha A, Hellgren E, Falkenhav M, Sackeya P (2011). Screening and treatment of problems after intensive care: A descriptive study of multidisciplinary follow-up. Intensive & Crit Care Nurs.

[25] Jones C (2014). Recovery Post ICU. Intensive & Crit Care Nurs.

[26] Åkerman E, Fridlund B, Ersson A, Granberg-Axéll A (2009). Development of the 3-SET 4P questionnaire for evaluating former ICU patients’ physical and psychosocial problems over time: A pilot study. Intensive & Crit Care Nurs..

[27] Oxford Learner’s Dictionaries. 2017-12-16. https://oxfordlearnersdictionaries.com/definition/english/recovery?

[28] Gadamer H-G. The enigma of health. Standford California:Stanford University Press. 1996;

[29] Eriksson K. Hälsans idé. [The idea of health]. 2^nd^ ed. Stockholm: Almqvist & Wiksell;1986.

[30] Eriksson K, Bondas-Salonen T, Herberts S, Lindholm L, Matilainen D (1995). Den mångdimensionella hälsan – verklighet och visioner. [The multidimensional health –reality and visions]. Slutrapport, pp 1–62. Vasa Sjukvårdsdistrikt SKN, Institutionen för vårdvetenskap.

[31] Bergbom I (2008). The process of recovery from severe illness, injury or surgical treatment. Rec Adv Research Updates.

[32] Ringdal M, Johansson L, Lundberg D, Plos K, Bergbom I (2009). Outcomes After Injury – Memories, Health-Related Quality of Life, Anxiety and Symptoms of Depression After Intensive Care. Journal of Trauma.

[33] Yaghmale F (2003). Content validity and its estimation. J Med Educ..

[34] Nunnally JC (1994). Bernstein, IH. Psychometric theory. 3rd ed. New.

[35] Fayers PM, Machin D. Quality of Life – The assessment, analysis and reporting of patient reported outcomes. 3^rd^ ed. Oxford: Wiley-Blackwell. 2016;

[36] Hays RD, Hayashi T, Carson S, Ware JE (1988). User’s guide for the Multi-trait Analysis Program (MAP).

[37] Cöster H. Att kunna tala allvar med sig själv. [To be able to seriously talk to yourself]. Karlstad: Karlstad University Studies; 2003:10.

[38] Eriksson K (2006). The suffering human being.

[39] Morse JM (1997). Responding to threats to integrity of self. Adv Nurs Sci.

[40] Ringdal M, Plos K, Bergbom I (2008). Memories of being injured and patients’ care trajectory after physical trauma. BMC Nursing.

[41] Ringdal M, Rose L (2012). Recovery after critical illness: The role of follow-up services to improve psychological well-being. Crit J Nurs Res..

[42] Pett MA, Lackey NR, Sullivan JJ (2003). Making sense of factor analysis: The use of factor analysis for instrument development in health care research.

